# Social Entrepreneurship Orientation and Enterprise Fortune: An Intermediary Role of Social Performance

**DOI:** 10.3389/fpsyg.2021.755080

**Published:** 2022-02-14

**Authors:** Zuhaib Zafar, Li Wenyuan, Mohammed Ali Bait Ali Sulaiman, Kamran Akhtar Siddiqui, Sikandar Ali Qalati

**Affiliations:** ^1^School of Management, Jiangsu University, Zhenjiang, China; ^2^Department of Marketing and Entrepreneurship, Dhofar University, Salalah, Oman; ^3^Mathematics and Social Sciences, Sukkur IBA University, Sukkur, Pakistan; ^4^School of Finance and Economics, Jiangsu University, Zhenjiang, China

**Keywords:** social entrepreneurship orientation, social enterprise, hybrid, mediation, financial performace

## Abstract

Social entrepreneurship orientation (SEO) is a behavioral construct of social entrepreneurship (SE); therefore, we examined the influence of SEO of the organization on social and financial performance. A random sample of 810 employees was drawn from social enterprises of Pakistan during the COVID-19 pandemic. Although increasing research focuses on SE, the discipline continues to disintegrate, and this has led to appeals for a careful investigation of the associations of firms’ SE. In the recent decade, “social entrepreneurship” has earned its importance as a segment of entrepreneurship. Instead of mixed activity, firms are more likely to engage in either for-profit or non-profit activities. The causes for and consequences of this conduct has been mainly studied using objective measures of SEO, social performance, and financial performance, with little attention paid to the subjective experiences of social enterprises. We rely on the theory of stakeholder and mixed structuring to postulate that social performance intermediates the SEO-financial performance relation. By assessing a sample of 810 employees from active enterprises, we discover that social performance mediates positively and partially between SEO and financial performance, and both direct and indirect paths are in the same direction and significant. Our findings exhibit that social performance variance explained 74% of the mediating role, and the remaining 26% of the effect is because of SEO. We consider the functions by which an SEO influences enterprise performance and delivers more prominent understanding into multiple spectrums of performance. We discuss the prospective suggestions of our research and foster an encouraging pathway for more enquiry on the SEO paradigm. The study adds contribution to the literature, which has not been testified before on hybrid firms. SEO is a newly defined construct and requires more prospective research. This research gives the researchers/scholars new directions to address related disciplines and further explore this domain.

## Introduction

Social entrepreneurship (SE) is awakening in interest and its literature is rapidly growing (Halberstadt and Kraus, 2016; Semrau et al., 2016; Sassmannshausen and Volkmann, 2018; Sutter et al., 2019). Over the past 10 years, SE research has definitely gained attention *via* generating communal value and encouraging societal change, mainly in the perspective of the developing economy (Rao-Nicholson et al., 2017; Del Giudice et al., 2019). Certainly, the latest scientific framework’s bibliometric inquiry for entrepreneurship research (Ferreira et al., 2019) discovered six fundamental entrepreneurship theories (1, innovation theory, 2, need for achievement theory, 3, theory of social change, 4, theory of social behavior, 5, theory of model personality, and 6, theory of leadership), which described the theory of SE as one of the six foundational elements of entrepreneurship research. Identifying that the growingly complicated environmental and socio-political context has allowed for a range of social issues, social entrepreneurship orientation (SEO) has appeared as challenging to normative to traditional commercial activities with the capacity to solve them. SEO incorporates the primary goal of having a communal impact, in which SEO actions pursue to deal with social challenges (Austin et al., 2006; Ramani et al., 2017; Lumpkin et al., 2018) to accomplish “valuable results from prosocial activities” valued by the envisioned objectives of that activity and/or the broader society of people, businesses, and/or contexts (Rawhouser et al., 2019). Scholars have studied SEO commonly and understandable by its “hybrid” quality, collaborating a societal pursuit with the entrepreneurial procedures and practices (Saebi et al., 2019). SEO is an entrepreneurial action that develops in a “hybrid” nature within enterprises because social value creation occurs *via* market-oriented techniques (Miller et al., 2012). Therefore, SEO comprises a mixed form, which can be defined as “actions through which companies create sense and mix (various) forms” (Battilana and Lee, 2014) or “the mixture of components of the company that conventionally would not have gone unitedly (Battilana et al., 2017). Formerly, scholars have hypothesized that companies mainly fulfill any of two purposes, one social or economic purpose (two pure forms that compete in a hybrid society) (Pache and Santos, 2013), and have paid attention to the balances between society and the economy preferences. These assumed balances could interrupt the provision of resources (Smith et al., 2013) and reduce the company’s competence (Fiol et al., 2009). We propose that SEO settles the hybrid balances (tensions) of social value formation and the acquisition of economic value and permits companies to respond to social and economic issues within society and in so doing and social and economic goals become harmonizing instead contending (Tobias et al., 2013).

A problem has been restricted as the concept of SEO has been derived from various fields and disciplines (e.g., entrepreneurship, economics, sociology, and ethics) (Saebi et al., 2019). Regarding its concept, the majority of inquiries to date have pursued to transmit entrepreneurial orientation into social perspectives to aid in explaining the concept of SEO (Santos, 2012; Guo and Bielefeld, 2014; Alarifi et al., 2019). Therefore, scholars have determined SEO in primary terminologies as non-profit entrepreneurial orientation (Dwivedi and Weerawardena, 2018). Still, the abstraction of SEO requires a more transparent picture; in addition, this restrains our conception of development and limits the emergence of collective understanding of SEO and constitutes a distinctive concept in its own right (Saebi et al., 2019). We discuss that such a methodology underemphasizes an SEO’s strategic properties and neglects to identify that the actions stimulated by an SEO are possibly apart from those causing an orientation widely concentrated on revenue (Kraus et al., 2017).

Regarding the organizational challenges in SE studies, the methodological difficulties of past research have supported the employment of related dimensions from the entrepreneurship fields to grow the SE discipline (Short et al., 2009). This covers the distinctiveness of the SEO concept (Lurtz and Kreutzer, 2017). Thus, the current capacities of the SEO concept are not sufficient and inadequately disclose the underlying processes of the SEO construct in contrast to other recognized aspects (Saebi et al., 2019). The research also strengthens the SEO concept and further formulates the use of the SEO construct.

In the recent decade, “social entrepreneurship” has earned its importance as a segment of entrepreneurship. Instead of mixed activity, firms are more likely to engage in either for-profit or non-profit activities. The causes for and consequences of this conduct have been mainly studied using objective measures of SEO, social performance, and financial performance, with little attention paid to the subjective experiences of social enterprises.

The process by which SEO affects financial performance is empirically supported in this study. We pursue a central base between the supported idea of employing theories from different fields (e.g., stakeholder theory and hybrid establishing view) and the requirement to obtain the distinct of the SEO construct to improve enclosure of the field of SE (Dwivedi and Weerawardena, 2018).

The SEO construct has already been justified by Kraus et al. (2017); the construct can be used for firms that target hybridity (social and financial goals). The research adds contribution to the literature, and that has not been testified before on hybrid firms. Mainly, how does SEO help enterprises achieve social performance? Besides, how do such enterprises manage to achieve their financial performance? And do these enterprises serve social performance while also achieving their financial goal?

In context of Pakistan, around 448,000 social enterprises are operational, and their contribution to GDP is high. Multi-sector enterprises are approximately divided into 53% education, 30% health and social care, 11% agriculture and fisheries, 9% energy and clean technology, 3% forestry, and 2% transport. In the prevailing legal framework, the choices for the certification of social enterprises lie in two large divisions: for-profit and non-profit and take numerous varieties. Available legal forms of for-profit social enterprises have been further categorized as 1, sole proprietorship, 2, association of persons/partners, 3, private limited companies, and 4, public limited companies (Ahmed et al., 2016).

## Theoretical Model

### Social Entrepreneurship Orientation

While the construct of SE has existed since the 1950s (Bowen, 1953), it has only received increasing attention in the last 10 years (Sassmannshausen and Volkmann, 2018; Saebi et al., 2019). The motives for this attention are on account of SE being revealed as:

• An influential process to fight grave poverty (Sutter et al., 2019).
• A motivation of transformation in social settings (Alvord et al., 2004).
• A dynamism intended for institutional change (Nicholls, 2008; Cajaiba-Santana, 2014).
• An essential determinant in promoting economic development and the growth of existing markets (Azmat et al., 2015).

Based on models of commercial and social businesses that differentiate businesses from exclusively hybrid, philanthropic, or profit-generated companies, SEO is different due to its communally inclined and revenue-oriented nature of entrepreneurship (Dees, 2001; Swanson and Zhang, 2011). SEO, as a mixed form of entrepreneurship within organizations, appeared owing to institutional gaps. Freshly developed organizations, hybrid businesses, rely on social entrepreneurial activities (i.e., SEO actions), which signify the conversion of already set up institutions in ways that will differ from the status quo (Maguire et al., 2004; Austin et al., 2006; Doherty et al., 2014).

The perspective of institutional entrepreneurship highlights that the shortage of financial support from the government and decrease of private aids affect the sustainability of non-profit organizations (NPOs) (Dart, 2004), and accordingly, the different nature of NPOs develops from the complicated social entrepreneurial activities of current institutions (Ko and Liu, 2021). An NPO is regarded as a mixed company that engages SEO (Fitzgerald and Shepherd, 2018), which is newly entrepreneurial, enterprise-oriented, market-inclined (Maier et al., 2016), social mission-compelled and pays attention to producing profits from commercial actions. In addition, the identity of such mixed enterprises is twofold and is shaped by the mixing of social and economic requirements (Moss et al., 2011). Companies mentioned earlier, which search to cover environmental and social challenges by participating in entrepreneurial activities, have been increasing in number. Several companies are now changing their attention to improve social and financial advantages (Nicholls, 2008). The complication falls in the possible tightness at the core of these hybrid companies between the societal and the financial.

The SEO is a multi-spectral concept in that (a) its aspects signify entrepreneurial actions (proactiveness, innovativeness, and risk-taking), and (b) it adds a dimension of social mission that fulfills the righteousness of an SEO (Weerawardena and Mort, 2006).

Socially active entrepreneurs take part in entrepreneurial activities such as modernism, opportunity recognition and utilization, and deployment of resources around a scientific solution (Ratinho et al., 2015). However, social entrepreneurial activities are used mainly in order to gain the social aim and in which the recognition of entrepreneurial revenue-producing likelihoods is derived from social issues (Ramani et al., 2017), which is mainly the inspiration for women entrepreneurs (Rosca et al., 2020). Therefore, social entrepreneurial actions distinctly take social value construction and financial profits (Bacq et al., 2016) in an implicit sequence that kicks off with the communal component.

In SE literature, the restrictive determinant in developing SEO is its heterogeneity. Various mechanisms are engaged in a similar theoretical hierarchy, for which they may not be part. This makes it challenging to match the multiple results related to SE literature (Saebi et al., 2019). Due to this heterogeneity, SE is not easy to capture and presents direct challenges in proceeding with the SEO construct in scholarly research. The issue is that researchers have not specified the form of SEO, and there is an unclear borderline with associated concepts. Differentiating SEO from associated constructs such as the employability of the concept “social entrepreneurship” is promptly escalating, there continues to be little agreement regarding its explanation (Austin et al., 2006; von der Weppen and Cochrane, 2012; Zahra et al., 2014; Halberstadt and Kraus, 2016; Rao-Nicholson et al., 2017). The SEO concept is, at its crux, a “contended construct” (Choi and Majumdar, 2014) and “there is no conclusive agreement to what the concept really intends” (Nicholls, 2010). Saebi et al. (2019) pointed out that “there is no correspond[ing] description and obvious dimensionalization of the social entrepreneurship paradigm.”

A comprehensive paradigm of SEO is required to emphasize the importance of the orientation used by enterprises and identify the significance of such an orientation (Short et al., 2009). To highlight this, we attempt to distinguish SEO from related concepts that are frequently referenced simultaneously in the research. SEO distinguishes itself from firm-level operations that are characterized through an economic purpose (commercial entrepreneurship) or entirely social goals (philanthropic/non-profit enterprises) (Saebi et al., 2019). We propose that SEO should be conceptualized uniformly to facilitate methodological improvement and help realistic measurement of the concept (Sassmannshausen and Volkmann, 2018).

The lines between NPOs and SEO have been unclear. NPOs strive for value to society to satisfy society’s needs as a whole, such as those who are needy (Certo and Miller, 2008). Whereas NPOs may produce income by participating in social tasks such as fundraising or gaining donations. Such profits are applied to a particular program lasting a specific amount of time and are not long-running or developed over time, which is a defining feature of entrepreneurial activities. According to behavioral entrepreneurship theory, we perceive SEO as a plan-of-action, psychological, and organizational construct. A vital component of entrepreneurial behaviors is that they have to be performed constantly and consistently in a repetitive manner in order to build an orientation (Covin and Slevin, 1991). As a result, this research contextually investigates the impact of entrepreneurial actions within social enterprises to affirm SEO. Besides, it seeks to establish a respective SEO definition and assesses its effect within enterprises whose income-producing practices hold a strategic longevity orientation with observable financial objectives (Saebi et al., 2019).

Furthermore, the distinctions between SEO and corporate social responsibility (CSR) have not been set, with some scholars defining SEO as traditional entrepreneurship with a CSR aspect (Surie, 2017). CSR is characterized primarily as social behaviors that help the community; however, CSR does not continually transform into inventive or entrepreneurial activity and typically symbolizes a firm’s societal responsibility (Shepherd and Patzelt, 2011). CSR begins with the company’s existing actions and then moves on to how they might be better targeted to users to generate revenue. On the other hand, SEO begins with identifying an unsatisfied social need, primarily indicating profitable opportunities (Zahra et al., 2014).

Similarly, whereas SE is regarded as an extension of the concept of entrepreneurship, it stretches literature beyond the limitations of conventional entrepreneurship (Dwivedi and Weerawardena, 2018; Saebi et al., 2019). Incorporating SEO among company-level operations has made it hard for researchers to distinguish SEO from entrepreneurial orientation, which has been measured using the EO scale. As a result, previous studies have remained unsuccessful in building a different definition of SEO to determine what actual benefits are fostered by SEO (Duvnäs et al., 2012; Miles et al., 2013). Duvnäs et al. (2012), for example, employed the Covin and Slevin (1989) scale to assess social innovation orientation, although these items primarily evaluate the innovation aspect of EO. Kuratko et al. (2017) also investigated this concept through piloting a SE scale by modifying their commercial entrepreneurship scale. A significant drawback is that several characteristics of the commercial entrepreneurship scale are insufficient to describe the specific context of SEO. It has increased the uncertainty of the SEO construct, and scholars have found that SEO is not a distinct concept from commercial entrepreneurship (Dacin et al., 2010). A conceptual gap for both SEO and commercial entrepreneurship must be established, and an SEO assessment scale is needed. SEO differs from typical entrepreneurship in that it combines entrepreneurial philosophy with a social objective (Leadbeater, 1997; Sullivan Mort et al., 2003; Weerawardena and Mort, 2006).

Specifically, Dees (2001) briefly discussed whether SE is linked to:

1. Acceptance of a vision to establish and maintain social value.
2. Identifying and persistently seeking new possibilities to support the objective.
3. Dedication to a continuous adaptation and learning process.
4. Operating beyond consideration for the resources that are already available.
5. Showing a stronger sense of social responsibility for the public reached by the organization and the outcomes it produces.

On the enterprise level, it is recommended that companies acquiring SEO intend to generate a social value as a path to broader wealth generation for the enterprise and society.

### Social Entrepreneurship Orientation and Social Performance

Performance may be assessed concerning financial values, usually employing a financial accounting system and/or regarding non-financial conduct (Hendriksen and Van Breda, 1999). Measuring performance with non-financial indices has increased its acceptance as a system of the social activity of companies (Oliveira et al., 2009). Social companies are institutions driven together by a social purpose and financial productivity that manages to balance the resources required to have social advantages. These firms meet some of the gaps between value found for-profit and non-profit businesses. For instance, some universities, colleges, hospitals, and other social institutions are structured as social firms (Miles et al., 2013).

Oriented behaviors of SE are distinguished through their mixture of societal missions with entrepreneurial activities to accomplish stability for mixed enterprises. These hybrid firms are being formed owing to government avoidance/unattainability and disinclination of the private segment to cover the unfulfilled social tasks. Its central core, SEO actions, engage social value creation (Chell et al., 2016). The concept of creating social value is a usual topic in descriptions of SE (Dacin et al., 2010). SEO’s social value proposition is described as SEO’s capability to “generate social value by encouraging communal transformation or fulfilling need of society” (Mair and Marti, 2006). Therefore, SEO’s social value proposition, its “steering axle,” presents a hybrid firm’s aimed promise, when using SEO, in giving importance to its recipients (Covin et al., 2015). At its core, it points out the value that social entrepreneurial activities provide for an object market (Kraus et al., 2014). The social effect of a mixed company depends on its capability to obtain advantages from SEO to its different recipients and maintain its social value offering to augment value for its planned objectives and ecosystem.

Social entrepreneurship orientation attends to achieve challenging objectives by assigning priority to the creation of social value over financial performance. The creation of social value discusses the organizational efficiency in dealing with social issues or issues that arrange a mixed (hybrid) company (Kroeger and Weber, 2014). It takes place when the diverse firm “accomplishes a comparable social return with little money or produces higher social welfare as good as cost” (Porter and Kramer, 1999). Mixed firms use SEO targets to accomplish their social purpose and thrive on producing social value when pursuing to sustain their practicality *via* returns that are made in an entrepreneurial and innovative manner (Certo and Miller, 2008). The creation of social value ensues from the organization’s decision-making actions and practices that engage behaviors of SEO and the search for new opportunities to suggest modern solutions to social provocations. In the perspective of mixed enterprises, we take an end-user context on social wellbeing and inquire the supposition that all human performance is only encouraged by self-interest and welfare. The aim of the mixed enterprise in using SEO is accomplishing social wellbeing, which stimulates the enterprise’s personnel to increase the scope of self-interests (Bridoux and Stoelhorst, 2016). This prevalent logic postulates as

**H1:** Social entrepreneurship orientation has an impact on social performance.

### Social Performance and Firm Financial Performance

To explicate the social-financial performance relation, we depend on stakeholder theory, a leading strategic method in the literature of social matters (Goldsby et al., 2018). Past studies have determined and inquired stakeholders’ attention from various dimensions (Donaldson and Preston, 1995), containing both a normative perception (i.e., clearing up why stakeholder relations would affect the enterprise) and a descriptive perception (telling how stakeholder relations are considered). We implement an instrumental technique (investigating the advantages of assuming the interests of stakeholders) within purposefully oriented hybrid enterprises. In which social performance is influential in advancing financial revenues (Jones, 1995), and based on the primary proposition that successful results from social productivity are linked with the degree to which the enterprise administers the welfare of its straight and broader range of stakeholders (Van der Laan et al., 2008).

According to instrumental stakeholder theory, socially conscious and receptive enterprises are smarter and more capable of negotiating complicated webs of stakeholder relationships (Rowley, 1997; Rahman et al., 2020). While hybrid organizations involved in real contexts with diverse participants (Hillman and Keim, 2001), improve their legitimacy (Suchman, 1995), build positive credibility (Orlitzky and Swanson, 2008), improve product–market-based efficiency (Rahman et al., 2020), and, essentially, increase their financial sustainability (Mahon, 2002). Husted and de Jesus Salazar (2006) developed a conceptual model that takes a strategical or operational method to impact social performance on financial performance. In this approach, strategically oriented hybrid enterprises gain profits from social investments (whether by generating “social goods” through scholarship provision or reducing “social bads”) (Rawhouser et al., 2019). Enterprises and markets are endowed with a wide range of values, not just economic value structures (Orlitzky, 2011). Markets are integrated with more extensive social processes (Whittington, 1992). Thus, enterprises must not lose perspective of larger societal objectives or might lose risk credibility (Suchman, 1995), particularly amongst progressively socially conscious clients (Rahman et al., 2020). However, in strategically focused hybrid enterprises, social entrepreneurial efforts are not philanthropic but are (in theory) purposeful and can improve or at least heighten financial performance (Saebi et al., 2019). A NPO may attempt to enhance social efficiency from social programs. Still, it does not intend to gain benefits from its social investments. Because of that, it is less likely to obtain monetary gains in contrast to a strategically oriented mixed organization (Husted and de Jesus Salazar, 2006).

In contrast, in a strategically focused hybrid enterprise, social investments would promote public welfare and result in new capacities, establishing the conditions for the organization’s financial performance to improve. The strategic case of corporate social investment demonstrates that social investments can be profitable for strategically inclined hybrid enterprises (Husted and de Jesus Salazar, 2006). Strategic hybrid enterprises market social innovations to support local communities (Del Giudice et al., 2019) and participate in entrepreneurial projects to be more approachable to capital markets (Nicholls, 2009). Orlitzky (2001) conducted a meta-analysis to validate the theoretical expectation outlined in the instrumental approach to stakeholder philosophy that financial output accumulates to those enterprises that best address the requirements of their stakeholders in broader society (Orlitzky, 2001; Santos, 2012). As a result of this prevalent reasoning,

**H2:** There is a positive relationship between social and financial performance.

### The Role of Social Performance in Mediating the Social Entrepreneurship Orientation-Financial Performance Relationship

The literature on diverse organizations has revealed the long-run effect of socio-economic tensions, addressing the peril of “mission drift” (deviance of a company from its destination), which can arise when the conciliation processes of conflicts in the management of the exchange between economic attainment convert into decisive activities that are inconsistent with the established strategic goals (Ebrahim et al., 2014). Regardless of the problems linked to integration hybrid tensions, another study has shown that social value generation and economic value capture are closely related in SE hybrids because the SEO firm removes hybrid organizational tensions to interlink the likelihood of greater performance or influence with greater financial profits (Tobias et al., 2013). This indicates that the social logic of referencing, imbued with enterprise-motivated behaviors, generates social and financial performance prosperities. Social and financial performance, therefore, may be assumed to become paired in the instance of SEO.

Social businesses are organizations focused together by a social drive and financial efficiency that accomplishes balance to articulate the resources required to gain social leads. These companies cope with some of the gaps between value established in for-profit and non-profit companies. For instance, some hospitals, universities, colleges, and other social bodies are organized as social businesses (Miles et al., 2013). Furthermore, other studies have shown that hybrid qualities are mutually reinforcing, in which companies respond to compound needs of people (Paolella and Durand, 2016), and become society-based enterprises (Peredo and Chrisman, 2006) and hybrid companies that create value *via* transformation of underused incompatible resources (Hockerts, 2015). A past study has also indicated opportunities for mutually reinforcing social and economic objectives (Battilana and Lee, 2014; Spieth et al., 2019). A mixed organization is more distinctive than formerly thought, and opportunities to generate higher social value do not essentially come at the cost of lower economic value (Shepherd et al., 2019). However, the tensions between economic and social modalities do not entirely disappear with SEO. We anticipate that the SEO contribution to financial performance may first depend on its capability to generate social performance. Comparative hybridization is measured based on the relative importance of business inclusion in economic and social logic (Shepherd et al., 2019).

The hybrid establishing literature has revealed the long-run effect of socio-economic apprehensions, addressing the possibility of “mission drift” (the deviation of an enterprise from its oriented objective), which can take place when clashing settlement procedures in maintaining the exchange between economic and social realization lead to organization engagements that are inconsistent with the defined strategic goals (Ebrahim et al., 2014). Despite the problems linked to the settling of mixed tenseness, another study has found that creation of social value and capture of economic importance are interwoven in SE mixes such that the company involved in SEO is estimated to eliminate the mixed construct tensions to interweave the likelihood of greater social productivity or influence with more incredible financial benefits (Tobias et al., 2013). This indicates that SEO’s social rationale, impregnated with enterprise-motivated undertakings, deduces social and financial performance profits. In that way, social and financial productivity may be anticipated to be integral in the instance of SEO. However, under SEO, the conflicts between civic and economic manners do not go away entirely. We expect that SEO’s participation in financial performance may be based primarily on its capacity to produce social performance. The relative significance of firms attributing to economic and social logic is used to determine relative hybridity (Shepherd et al., 2019). Non-profit businesses have a higher level of social sense (lower hybridity) than hybrid enterprises that use SEO conducts. Still, traditional for-profit firms have a higher level of economic rationale (also inadequate in hybridity). SEO has a high degree of relative hybridity, and it entails the co-creation of entrepreneurial possibilities for the benefit of both the community and the company (Alvarez and Barney, 2010; Venkataraman et al., 2012). SEO operations bridge the space between economic and social value logic when businesses participate in SEO and follow potential solutions that create an excellent social effect and company monetary gains. As a result, companies that use SEO will see an improvement in relative hybridity. Although not all social businesses with a leading social logic are entrepreneurial, only when they utilize SEO practices, do they get closer to mixed types of companies and may employ SEO behaviors to address contradictions between social and economic value. Contrasting with an EO, which is based on a pure commercial and institutional logic established with the aim of value creation (Austin et al., 2006; Schneider, 2017), SEO is less straight in that it is not financially pushed and is not focused on the pure institutional logic of aiming financial profits for shareholders, but has been linked to productive financial profits for hybrid enterprises (Mair et al., 2015).

**FIGURE 1:**
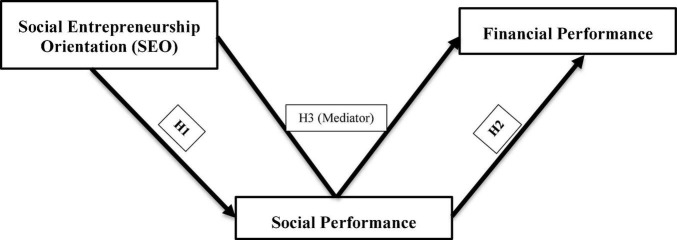
Conceptual model.

Efficiently navigating the coexistence of social wellbeing and economic logic, the organizational behavior must align so that various institutional logics are integrated and balanced (Battilana and Lee, 2014). Compared to other organizational processes like EO, SEO can handle the coexistence of social and economic logic within businesses by balancing the two. Communally entrepreneurial activities provide double value, not only a social advantage that is critical to a company’s competitive position (Alter, 2004; Porter and Kramer, 2011; Ridley-Duff et al., 2016). SEO practices demonstrate a mixed value, indicating that each company may gain several forms of benefit (i.e., financial, social, environmental, etc.). SEO is defined as the “recognition, creation, assessment, and utilization of options to build innovative enterprises, frameworks, and solutions with an emphasis on generating mixed value,” according to Zahra et al. (2014). Scholars have identified several possible SEO benefits, such as increased efficiency, increased market share, and a long-term competitive edge (Porter and Kramer, 2011; Fellnhofer et al., 2014). The development of social value is interrelated to the generation of economic wealth, as per the social value proposition of SEO (the differentiating core idea of SEO) (Dees, 2001; Hlady-Rispal and Servantie, 2018). Due to the strategic intention of participating in societal entrepreneurial initiatives that would generate value for the business, substantial financial performance is predicted by a mixed firm led by SEO practices. Lasting long-run value capture is reliant on the creation of social value (Dees, 2001). We anticipate that SEO strategically accomplishes superior social performance, resulting in economic benefit (Weaver et al., 1999; Cornelius et al., 2008; Santos, 2012; Guo and Bielefeld, 2014). Our proposed model is depicted in Figure 1. SEO provides social value for establishing new markets and meeting unmet social demands, creating economic gain (Ramani et al., 2017). Companies progressively realize the advantages of producing social value generation explicitly and a goal in itself, instead of a spin-off of firm-level behavior (Zahra et al., 2009). As this social value-generating behavior can take place in various industries or sectors (Austin et al., 2006; Arribas et al., 2012). SEO-focused companies are characterized by their hybrid organizational structures and their capability to understand and utilize opportunities for more excellent value to society and economic benefits (Reis and Clohesy, 1999; Shepherd et al., 2019). Thus,

**H3:** Social performance plays a mediating role between SEO and financial performance.

## Methodology

One of the studies conducted by Ahmed et al. (2019), stated that social enterprises exist in Pakistan and they are actively working. Pakistan has seen an increase in the number of social enterprises working in diverse areas to address particular pressing challenges that communities face. Rapid urbanization, the development of public sector universities, the addition of more incubators, and speedup initiatives have all contributed to the emergence of a new generation of socially conscious entrepreneurs across the country. It is progressing through entrepreneurs who launch and implement initiatives in various fields, including energy, drinking water, education, health, construction, financial inclusion, and commercial, among others (Ahmed et al., 2019). Despite the fact that their total numbers are unrepresentative, they have been categorized into three major classes as for-profit, non-profit, and mixed (hybrid firms) (Ahmed et al., 2016).

### Sampling

In an investigational research, determining the optimal sample size is critical for minimizing sampling error (Hair, 2010). As a result, we chose statistical power assessment for this purpose (Cohen, 1988; Urbach and Ahlemann, 2010). Researchers recommend a higher sample size (Umrani et al., 2018; Hair et al., 2019; Muskat et al., 2019). As a statistical approach for determining the appropriate sample size for a specific research endeavor, power analysis is recommended (Saebi et al., 2019). As a result, we employed the G*Power 3.1 software to perform *a priori* power analysis technique for determining the minimum sample size for the current investigation (Faul et al., 2009; Hair, 2014). A minimum sample of 619 was extracted to be necessary applying parameters such as power (1 – β err probability; 95%), an alpha significance level (α err probability; 5%), medium effect size f2 (0.2), number of groups = 4, non-centrality parameters (λ = 24.8), and the number of predictors in our model (Cohen, 1992; Faul et al., 2009; Hair, 2014). Power analysis proposed that 619 samples must be obtained (extracted from G*power) to overcome the issue of inadequate response, therefore we needed to go up to an 810 sample size.

### Data Collection Procedure

For testing the hypotheses, a sample of 810 employees from active enterprises operating in Pakistan was used. We used an online survey method to gather data from these social enterprises. The present study asked employees of enterprises to fill in a multi-item survey. These employees were considered key informants and apt in terms of knowledge; also, they could provide accurate responses (Mubarik et al., 2016).

The study used a significant sample size (810 responses), and an online response was acknowledged; a Google form was sent to these enterprises to complete the sample. A sample of 810 was received from participants with no missing value, and all 810 cases were utilized to investigate the study. A total of 597 men and 213 women participated in this research, 73.7 and 26.3 %, respectively. Key informants ranged in age from 20 to 30 (291), 31 to 40 (366), 41 to 50 (97), 51 and higher (56) under percentages of 35.9, 45.2, 12.0, and 6.9, correspondingly. Career level varying from entry level (240, 29.6%), intermediate level (455, 56.2%), and high level (115, 14.2%) was included in this study. Likewise, in education level, participants were (278) intermediate, Bachelor’s (378), and Master’s or higher (154) degree holders with 34.3, 46.7, and 19.0%, respectively. Experience indicated that informants had different exposures such as less than a year (225), 1–5 years (382), 6–10 years (111), and 11 or greater (92) with percentages 27.8, 47.2, 13.7, and 11.4, respectively, as demonstrated in Table 1.

**TABLE 1:** Respondents’ characteristics.

Respondents characteristics	Frequency	Percent
**Gender**		

Male	597	73.7
Female	213	26.3
Total	810	100

**Age**		

20–30 years	291	35.9
31–40 years	366	45.2
41–50 years	97	12.0
51 or higher	56	6.9
Total	810	100

**Career level**		

Entry	240	29.6
Intermediate	455	56.2
High	115	14.2
Total	810	100

**Education**		

Intermediate	278	34.3
Bachelor’s	378	46.7
Master’s or higher	154	19.0
Total	810	100

**Experience**		

Less than 1 year	225	27.8
1–5 years	382	47.2
6–10 years	111	13.7
Greater than 11 years	92	11.4
Total	810	100

Table 2 presents the total of 810 employees who participated from active enterprises, 494 participants were from education with 61%, the health and care enterprises ratio was 29.1% with 236 participants, and the other services type enterprises ratio was 9.9% with 80 participants. Participants ranged in enterprises size including 5–30 (276), 31–60 (332), 61–99 (154), 100 or greater (48) under percentages of 33.1, 40.9, 19.1, and 5.90, respectively. Another enterprise parameter was age, 195 participants were from companies aged 1–5 years, 390 from companies aged 6–10 years, 162 from companies aged 11–15 years, and 48 from companies aged 15 years or higher with 24.1, 48.1, 20.0, and 7.80%, respectively.

**TABLE 2:** Enterprise information.

Enterprise characteristics	Frequency	Percent
**Enterprise type**		

Education	494	61.0
Health and care	236	29.1
Other services	80	9.9
Total	810	100

**Enterprise size**		

5–30	276	34.1
31–60	332	40.9
61–99	154	19.1
100 or higher	48	5.90
Total	810	100

**Age of enterprise (years)**		

1–5	195	24.1
6–10	390	48.1
11–15	162	20.0
15 or higher	63	7.80
Total	810	100

The data were collected during COVID-19, so the 810 responses took a long time. Due to the larger sample size, a major concern was how to deal with non-response bias, therefore an independent samples *t*-test was executed and followed as Armstrong and Overton (1977) suggested. Particularly, late and early respondents were divided into two groups (early and late respondents). It was assumed that late respondents were similar to non-respondents (Armstrong and Overton, 1977).

Independent samples *t*-test results indicated that no significant difference was found between the two groups, and the questionnaire was equally treated for the two groups. Resultantly, the study was free from the major concern of non-response bias. Besides, the study used a self-reporting scale in relation to Harman’s single factor test to ensure the absence of common method bias (Podsakoff and Organ, 1986). Peculiarly, EFA (exploratory factor analysis) was followed to evaluate the unrotated factor solution, and also the number of factors. Three factors were extracted from factor analysis, eigenvalues more than 1 and variance explained by the first factor occurred in 33%. Therefore, the current study did not find any common method bias issue.

### Measures

#### Social Entrepreneurship Orientation

A SEO questionnaire was utilized in this study, established by Kraus et al. (2017). The SEO construct was derived from four dimensions, namely, 1, social innovativeness, 2, social risk taking, 3, social proactiveness, and 4, socialness. Questions were surveyed on a Likert scale of five points, ordering from 1 (strongly disagree); 2 (disagree); 3 (neutral); 4 (agree); to 5 (strongly disagree).

#### Social Innovativeness

Social innovativeness is a sub-dimension of SEO; the scale of social innovativeness comprises three questions, originally developed by Kraus et al. (2017). A sample question, shown as, e.g., “Social innovation is important for our company,” was asked on a five-point Likert scale where 1 indicated strongly disagree and 5 strongly agree.

#### Social Risk Taking

Social risk taking is another dimension of SEO, the scale of social risk taking comprises three questions, originally developed by Kraus et al. (2017). A sample question, shown as, e.g., “Bold action is necessary to achieve our company’s social mission,” was asked on a five-point Likert scale where 1 indicated strongly disagree and 5 strongly agree.

#### Social Proactiveness

Social proactiveness is a dimension of SEO, the scale of social proactiveness consists of three items, firstly established by Kraus et al. (2017). An example question, presented as, e.g., “Our organization has a strong tendency to be ahead of others in addressing its social mission,” was asked on a five-point Likert scale where 1 indicated strongly disagree and 5 strongly agree.

#### Socialness

Another aspect is the socialness of SEO; the scale of socialness consists of three items, initially publicized by Kraus et al. (2017). An example question, presented as, e.g., “The objective to accomplish our social mission precedes the objective to generate a profit,” was asked on a five-point Likert scale where 1 indicated strongly disagree and 5 strongly agree.

#### Social Performance

The scale of social performance consists of four questions, primarily proven by Eggers et al. (2013) and Baker and Sinkula (2009). An example question is displayed as e.g., “Our organization is on a good path to accomplish its social mission.” Respondents were given a five-point Likert scale to rate the questions ranging from 1 = strongly disagree to 5 = strongly agree.

#### Financial Performance

The scale of financial performance contains four questions, primarily confirmed by Eggers et al. (2013) and Baker and Sinkula (2009). An example item is exhibited as, e.g., “In the past five years we achieved a higher profit growth than our (direct/indirect) competitors,” respondents were given a five-point Likert scale to rate the questions ranging from 1 = strongly disagree to 5 = strongly agree.

With respect to the adoption of the SEO and adaption of social and financial performance scales, another challenge was that this is the first study on enterprises in the Pakistani context; hence, in accordance with Tavakol and Dennick (2011), utilization of questionnaires in a study must be tested to assure their consistency in aiding to accomplish the research objectives. Particularly, it supports examining the efficiency of the scale to evaluate the constructs of the research. In addition, it aids to measure reliability and checks the consistency of every item on the scale. As per Sekaran (2009), reliability coefficients less than 0.60 reflect inadequacy. Nevertheless, the alpha coefficients extracted from the pilot testing fulfilled reliability criteria to apply the tool as all the constructs ensured alpha coefficients were higher than 0.7.

## Results and Discussion

The current research utilized PLS path modeling for analysis of data because this method is being recognized in the wide-range application of academic research (Lee et al., 2011; Hair et al., 2012). Initially, some assumptions were taken into consideration, such as multicollinearity, normality and common method variance were examined (Podsakoff and Organ, 1986; Tabachnick et al., 2007; Hair, 2010) then the researcher started analysis of reliability, validity, and structural paths. The current research engaged a two-step procedure, the first one was measurement model assessment, and the second was structural model assessment, for assessing and summarizing the results of PLS-SEM (Partial Least-Squares Structural Equation Modeling) (Henseler et al., 2009; Hair, 2010, 2014).

### Measurement Model Assessment

For assessment of the measurement model, as per Hair (2010, 2014) and Henseler et al. (2009), researchers need to calculate individual item reliability, internal consistency, convergent validity, and discriminant validity. PLS-SEM is best suited for the study because it has received scholars’ attention in various fields and is known for its wide acceptability, also PLS-SEM has a set of new standards for critical data analysis (Hair et al., 2019).

#### Individual Item Reliability

Individual item reliability should be evaluated by computing factor loading of every item under a construct (Hulland, 1999; Duarte and Raposo, 2010; Hair et al., 2012). Hair et al. (2019) suggested that a value of 0.6 or greater is considered acceptable for retaining an item. The current study reported all outer loadings were sufficiently higher than 0.5 values (see Table 3); thus, the study fulfilled the criterion of individual item reliability.

**TABLE 3:** Mean, standard deviation, Cronbach alpha, composite reliability, and average variance extracted.

Constructs	Mean	SD	CA	CR	AVE
Social innovativeness	3.44	1.07	0.871	0.920	0.794
Social risk taking	3.50	1.17	0.869	0.920	0.793
Social proactiveness	3.51	1.13	0.882	0.927	0.810
Socialness	3.41	1.16	0.821	0.893	0.736
Social performance	3.37	1.17	0.915	0.940	0.798
Financial performance	3.18	1.32	0.940	0.957	0.847

*SD, standard deviation; CA, Cronbach alpha; CR, composite reliability; AVE, average variance extracted.*

#### Internal Consistency

A rule of thumb provided by Bagozzi and Yi (1988) and Hair et al. (2011, 2019) for determining coefficients of composite reliability recommended a cutoff of 0.7 or higher. Table 3 shows the coefficients of composite reliability for each construct in this study. As shown in Table 3, the coefficient of composite reliability for every of the construct fell in the range of 0.893–0.957; this recommends the adequacy of constructs’ internal consistency reliability (Bagozzi and Yi, 1988; Hair et al., 2011). The study reported variance inflated factor (VIF) that measures common method bias and collinearity. VIF is reciprocal of tolerance (Ringle et al., 2015) suggested a threshold of VIF as a value equal to or lower than 5 (Tables 3, 4).

**TABLE 4:** Factor loadings, variance inflated factor, and tolerance.

Construct	Item	Loading	VIF	Tolerance
**Social entrepreneurship orientation**				
**Social innovativeness**				
	Social innovativeness 1	0.906	2.498	0.400
	Social innovativeness 2	0.872	2.191	0.457
	Social innovativeness 3	0.896	2.277	0.439
**Social risk taking**				
	Social risk taking 1	0.859	1.937	0.516
	Social risk taking 2	0.898	2.570	0.389
	Social risk taking 3	0.915	2.795	0.358
**Social proactiveness**				
	Social proactiveness 1	0.873	2.162	0.463
	Social proactiveness 2	0.901	2.620	0.382
	Social proactiveness 3	0.924	3.112	0.321
**Socialness**				
	Socialness 1	0.846	1.591	0.629
	Socialness 2	0.859	2.066	0.484
	Socialness 3	0.868	2.151	0.465
**Social performance**				
	Social performance 1	0.924	4.245	0.236
	Social performance 2	0.911	3.589	0.279
	Social performance 3	0.897	3.212	0.311
	Social performance 4	0.839	2.070	0.483
**Financial performance**				
	Financial performance 1	0.932	4.437	0.225
	Financial performance 2	0.910	3.401	0.294
	Financial performance 3	0.918	3.641	0.275
	Financial performance 4	0.921	3.780	0.265

*VIF, variance inflated factor.*

#### Convergent Validity

Fornell and Larcker (1981) recommended that convergent validity be assessed through AVE (average variance extracted). However, following Chin (1998), a value of 0.5 or higher is acceptable to represent the convergent validity of a specific variable. The AVE values given in Table 3 demonstrated that all the variables of this study met AVE above the threshold of 0.5; therefore, it is determined the study indicated adequacy in convergent validity (Chin, 1998).

#### Discriminant Validity

According to Fornell and Larcker (1981), a criterion to use a value of AVE 0.5 or higher, as a rule of thumb, can assess discriminant validity. In addition, it is recommended that the AVE’s square root should be greater than the correlations among the latent constructs for determining discriminant validity. Table 3 shows that the AVE values of all latent variables were higher than the cutoff. Table 5 shows that AVE’s square root was greater than the correlations among the latent constructs. Hence, all measures conclude adequacy in discriminant validity for the present study.

**TABLE 5:** Discriminant validity.

Constructs	1	2	3	4	5	6
Financial performance	*0.920*					
Social innovation	0.401	*0.891*				
Social performance	0.754	0.440	*0.893*			
Social proactiveness	0.450	0.700	0.455	*0.900*		
Social risk taking	0.460	0.810	0.481	0.825	*0.891*	
Socialness	0.521	0.590	0.613	0.794	0.761	*0.858*

*Italic values indicate square root of AVE.*

### Structural Model Assessment

As per recent studies, *R*^2^ evaluates the predictive power of the model (Sarstedt et al., 2014). Cohen (2013) put forward that 0.25 is weak, 0.5 is moderate, and 0.75 is substantial; in our study, social performance = 0.305 and financial performance = 0.581 in the coefficient of determination, as shown in Table 6. The residual standardized mean square root is the absolute standard of fit, and a value of zero shows a perfect fit. The SRMR is referred to as “the mean square of the difference between the observed correlations and the correlations implicit in the model.” The outcomes show a significant value of SRMR = 0.076 and NFI = 0.830; the NFI estimate was lower than the suggested value of 0.9 but greater than 0.8 indicates acceptable fit (Zikmund, 2003). If the SRMR value is less than 0.08, it is generally reflected as a good fit (Hu and Bentler, 1998). This study provides and guarantees the quality of fit, see Table 6.

**TABLE 6:** Model fit summary.

Sample (*N*)	810	*R*^2^ for social performance = 0.305
SRMR	0.076	
d_ULS	1.206	
d_G1	0.736	*R*^2^ for financial performance = 0.581
d_G2	0.581	
Chi-Square	2,649.98	
NFI	0.830	

*SRMR, Standardized Root Mean Squared Residual; d_ULS, the squared Euclidean distance; d_G, geodesic distance; NFI, normed fit index.*

**FIGURE 2:**
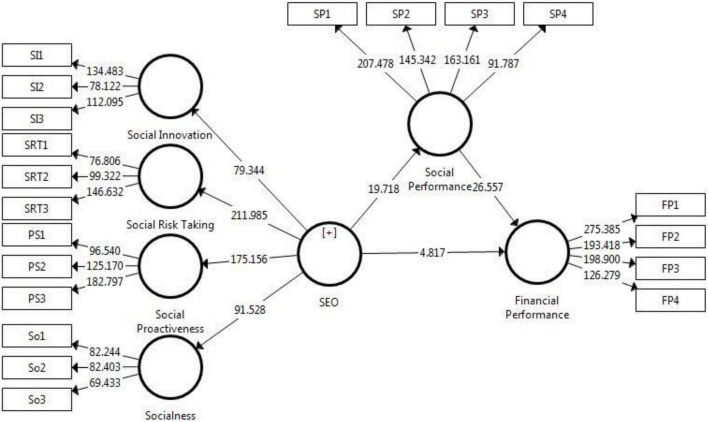
Hypothesis results.

The current research executed the bootstrapping method with 5,000 bootstrap samples and an 810 sample size to examine the path coefficients and their quality of being significant according to Hair (2010, 2014), Hair et al. (2011), and Henseler et al. (2009). Under this structural model with statistics, full estimates were obtained as shown in Table 7 and Figure 2. First, H1 suggested that SEO will be positively associated with social performance. Results shown in Table 7 and Figure 2 acknowledged a positive relation between SEO and social performance at a 1% significance level (β = 0.552, *t* = 19.72, *p* < 0.00). Therefore, affirming H1.

**TABLE 7:** Structural model.

Hypothesis	Relationship	Beta	SE	*t*-Value	*p*-Value	Decision
H1	SEO → social performance	0.552	0.028	19.718	0.000	Supported
H2	Social performance → financial performance	0.681	0.026	26.557	0.000	Supported
H3	Mediating effect					
Direct effect	SEO → financial performance	0.133	0.028	4.817	0.000	Supported/partial mediation
Indirect effect	SEO → social performance → financial performance	0.376	0.023	16.599	0.000	
Total effect	SEO → financial performance	0.509	0.028	18.033	0.000	

In hypothesis 2, social performance impacts financial performance positively. Social performance and financial performance show a positive correlation, as seen in the results with a coefficient of the relationship of β = 0.681, *t* = 26.56, and *p* < 0.00. H2 was also supported.

The third hypothesis, social performance plays a mediating role between social and financial performance, reflects mediating effect (indirect effect); the result showed the positive SEO–social performance–financial performance relationship (β = 0.376, *t* = 15.6, *p* < 0.00), and supported H3.

According to Baron and Kenny (1986), if an indirect path passes through the intermediary construct (mediator), its significant relation indicates that the mediator plays its role between independent and dependent constructs. If the direct path from independent variable to dependent variable is significant, and an indirect path is also significant, this suggests partial mediation. If a direct path is insignificant and indirectly significant, this refers to a full mediation. Hence, the study achieves the partial mediating effect of social performance that supports hypothesis 3.

Although the work of Baron and Kenny (1986) continues to be debatable, the further mediation tests described by Zhao et al. (2010) provide a decision-tree and a step by step process for checking mediation, categorizing its nature, and understanding the suggestions of conclusions for theory construction and upcoming investigation. Zhao et al. (2010) also indorsed the concept of mediation types; complementary mediation is achieved when direct and indirect paths are significant and positive. This study achieves the results, see Table 7.

It is essential to mention that to decide the degree of the indirect effect, the variance accounted for (VAF) formula was employed as recommended by Hair et al. (2016). This method supports defining to what amount exogenous constructs openly describe the variation of the endogenous construct and how much of that variance is characterized by the indirect relationship *via* the mediator construct. The subsequent method portrays how the VAF was computed:


$$V\,A\,F=\frac{I\,n\,d\,i\,r\,e\,c\,t\,e\,f\,f\,e\,c\,t}{T\,o\,t\,a\,l\,e\,f\,f\,e\,c\,t}=\frac{0.376}{0.509}=0.74$$

### Discussion

This study set out to investigate the path from SEO to financial performance in the broader scale through first-hand research. Our results deliver an originally empirical breakthrough for the SEO construct. The findings advocate that the relationship between SEO and financial performance is objectively positively intervened by the social performance so that social performance surely mediates the relation between SEO and financial performance. SEO averagely contributed to better financial performance; however, its influence on social performance led to better financial performance (Santos, 2012; Guo and Bielefeld, 2014). The straight consequences were consistent with prior studies in the literature, which found that entrepreneurial action in the social perspective positively affects social performance.

On the other hand, prior studies have reported a direct negative effect on financial performance, as one study reported by Miles et al. (2013). Our outcomes show crucial new support to the research of SEO and SE among enterprises in a broad spectrum. Between SEO and financial performance, the mediation check takes social performance into account because a mediator tells the whole story of our empirical study. There is a positive indirect effect of referencing through social performance on the financial performance of the company. A business with a strong SEO focus can have superior social benefits and reap financial rewards by working parallel on social contributions. The firm does not suffer from the financial drawbacks of high SEO. We discover that the total effect is diverse from zero. In this logic, the mediated positive impact between SEO on financial performance through social performance does pay off for the direct positive impact of SEO on financial performance.

On the basis of these findings, using the VAF, it can be determined that the social performance variable in the current study model acted the part of the mediating construct between the SEO and financial performance, as 74% of the effect of the SEO on financial performance is clarified by the mediation of the social performance. As the VAF is higher than 20% but smaller than 80% reveals partial mediation, recommended by Hair et al. (2016); thus, this condition can be ordered as a partial mediation (Hair et al., 2016).

We therefore perceive that social performance is complementary for the companies that support SEO. However, the indirect effect (hypothesis 3) compensates for the direct positive effect that we discovered during the data analysis. This complementary effect has identified and represented an essential contribution to our understanding of SE and the implications of SEO in companies, and the consequence of the hybrid approach. This effect applies to all companies in our dataset, not just those with high SEO. A company with a comparatively low SEO still has a positive association between both performances. One likely reason is that hybrid companies comply with a business logic (trying to increase financial returns for shareholders), which compromises the hybrid company’s authenticity (to look more like a for-profit company) and its performance through economic activities (for example, Shepherd et al., 2019; Spieth et al., 2019). Hybrid companies are considered responsible to a different set of stakeholders. They must balance the expectations of numerous stakeholders, and through disregarding their social effect, mixed (hybrid) companies run the risk of probing the legitimacy of the social determination of stakeholders (Ebrahim et al., 2014). A high level of SEO achieves a significant social return, which escalates the financial profit, as hypothesized. Nonetheless, surprisingly, we uncovered new nuances in this connection, displaying that the financial achievement from social performance influences the cost of financial performance from high SEO. This reconfigures the relation between social performance and financial performance and between SEO and financial performance.

However, it is cautioned that this connection should be interpreted strictly as VAF identified, as this study classifies supplementary potential benefits for a social approach or environmental strategy, comprising the product market level and customer responsiveness (Rahman et al., 2020), shareholder value (Hillman and Keim, 2001), lawfulness by being authorized (Suchman, 1995), and capacity-building (Orlitzky and Swanson, 2008). There is an urgent need for academics to observe SEO concerning multiple forms of business performance to establish the severity of its positive and complementary properties.

Our results also make three further assistances. First, we disclose that SEO holds a positive influence on social performance with respect to the social value intention of SEO (Mair and Marti, 2006; Covin et al., 2015). SEO can produce a positive communal effect *via* orienting business activities to a social mission, generating social welfare, and playing a benevolence role (Saebi et al., 2019). Second, we make it known that social performance holds a confident impact on financial performance according to the theory of instrumental stakeholders (Orlitzky, 2001), where achieving greater societal performance is needed to develop financial performance in hybrid companies. The positive socio-financial performance results strengthen past studies (Orlitzky, 2001, 2011; Husted and de Jesus Salazar, 2006; Santos, 2012).

Moreover, in the third contribution, we disclose that social performance mediates the relation between SEO and financial performance. Mutually, we add to the literature on hybrid organizations by enlightening that SEO expands a company’s financial performance. It settles some of the hybrid tensions of numerous established judgments (Shepherd et al., 2019) in the setting of our conclusion of complementary judgment. SEO arranges social mission over economic profits, but its social mission superiority may come at the cost of greater direct economic revenues balanced by financial performance to the business’s financial performance. A crucial inquiry for researchers currently is how further to amplify this balance. SEO, by fulfilling its social mission, can catch value for the company. SEO is an activity at the company level that is distinguished by the combination of different institutional senses (institutional variety) in a business that can attain modern income-producing solutions for large and complex social difficulties (Santos et al., 2015).

Depending on the critical properties of SEO on financial performance only would unmask the essential process by which SEO positively involves the company’s financial performance. This helps to upsurge the SEO literature by revealing the influences of SEO and its indirect path to a company’s financial performance. We disclose that SEO works by a diverse path to financial performance through social performance. SEO intends to succeed in its social mission *via* entrepreneurial societal and revenue-earning actions.

## Conclusion

The SEO construct in nature is behavioral in that it describes the management propensity to entrepreneurial action (symbolizing the “how” of entrepreneurial conduct) and is a specific concept in which SEO impregnates social sense with economic activity. Evaluating the influence of SEO on business results remains a challenge; however, our research is the first step and an indication for upcoming researchers to establish SEO as a distinct construct and legitimize it in entrepreneurship research. An important matter that deserves more focus among academics is inspecting mediation mechanisms that help up-to-date management trends to social enterprise conducts in generating and apprehending value for enterprises. We disclose that the mediation mechanism that catches the financial value from SE activities is social performance. Although implications are real-time and based on the data gathered, COVID-19 might have affected the implications.

### Theoretical Implications

By means of stakeholder theory and hybrid structuring as a theoretical view, we empirically postulated and found that SEO positively affects financial performance *via* its impact on social performance. Our conclusions improve the entrepreneurship discipline by shifting the discussion to the intermediating mechanisms in the relation between SEO and financial performance. Our research is one of the scarce studies investigating the impact of SEO and its mediating pathway on financial performance in a big-scale empirical framework. This study on SE is still comparatively new, engaging open-ended exploratory investigation and acquiring a predominantly derived methodology (Cheah et al., 2019). The study achieved its significance as defined in the theoretical model, hypotheses are aligned with research objectives and research objectives are lined up with research questions.

### Practical and Managerial Implications

Most research focuses on the individual degree of assessment; enterprise-wide studies in the field of SE are scarce. However, we need to be attentive to how important it is to understand the intermediate circumstances through which an SEO produces revenues to gain business performance, and when it does not. The results have vital practical implications for managers. Our findings disclose that having a communally entrepreneurial orientation is valuable for the company’s social performance. If the company succeeds in its social performance, it also gains its financial goal (while intervening impact). Put differently, success leads to success. As a result, if an executive is enthusiastic regarding the business’s social impact or social benefit, they will also think of greater financial success for the company. Our further checks show that SEO directly affects financial performance positively. Besides, a positive effect of social performance on financial performance was seen. This study suggests that managers should invest in SEO that ultimately achieves financial performance. Intermediary factors are essential in understanding when, how, and why SEO adds financially to businesses.

### Limitations and Future Work

While this study delivers stimulating theoretical, conceptual, and empirical information on SEO, the results are viewed with certain limitations. Initially, this research concentrated on a sample of mostly Pakistani small-medium businesses. To improve the generalization of our results, future investigations can examine our model with samples from diverse perspectives, like developing or emerging markets and more prominent companies.

In addition, this study used indicators of perceptual performance. Prospective research may broaden our conclusions to contain target measures of performance. In conclusion, there are apparent divergences in why and how social enterprises are formed and managed, for example, by women (Rosca et al., 2020). Therefore, it could also be a stimulating prospect for research.

Despite these limitations, the current study originally theoretically examines a newly established SEO scale (Kraus et al., 2017) and disclose its effect on financial performance. As social impact remains to progress in importance in societies, enterprises, economies, and research, our research leaves a stable foundation for further study on SEO. Another limitation is that the COVID-19 pandemic was observed during data collection, future research results/implications may deviate from our research.

## Data Availability Statement

The raw data supporting the conclusions of this article will be made available by the authors, without undue reservation.

## Ethics Statement

The studies involving human participants were reviewed and approved by the Ethics Committee of Jiangsu University. The participants provided their written informed consent to participate in this study.

## Author Contributions

ZZ, LW, and MB contributed to the conception and design of the study. KS, SQ, and ZZ contributed to data acquisition and analysis. ZZ and SQ performed the study. LW supervised and helped to finalize the manuscript.

## Conflict of Interest

The authors declare that the research was conducted in the absence of any commercial or financial relationships that could be construed as a potential conflict of interest. The reviewers MMu and MMo declared a shared affiliation with several of the authors ZZ, LW, and SQ to the handling editor at time of review.

## Publisher’s Note

All claims expressed in this article are solely those of the authors and do not necessarily represent those of their affiliated organizations, or those of the publisher, the editors and the reviewers. Any product that may be evaluated in this article, or claim that may be made by its manufacturer, is not guaranteed or endorsed by the publisher.
